# Inferring Proteolytic Processes from Mass Spectrometry Time Series Data Using Degradation Graphs

**DOI:** 10.1371/journal.pone.0040656

**Published:** 2012-07-17

**Authors:** Stephan Aiche, Knut Reinert, Christof Schütte, Diana Hildebrand, Hartmut Schlüter, Tim O. F. Conrad

**Affiliations:** 1 Department of Mathematics and Computer Science, Freie Universität Berlin, Berlin, Germany; 2 International Max Planck Research School for Computational Biology and Scientific Computing, Berlin, Germany; 3 Institute of Clinical Chemistry, University Medical Center Hamburg-Eppendorf, Hamburg, Germany; University of Cantebury, New Zealand

## Abstract

**Background:**

Proteases play an essential part in a variety of biological processes. Besides their importance under healthy conditions they are also known to have a crucial role in complex diseases like cancer. In recent years, it has been shown that not only the fragments produced by proteases but also their dynamics, especially *ex vivo*, can serve as biomarkers. But so far, only a few approaches were taken to explicitly model the dynamics of proteolysis in the context of mass spectrometry.

**Results:**

We introduce a new concept to model proteolytic processes, the *degradation graph*. The degradation graph is an extension of the cleavage graph, a data structure to reconstruct and visualize the proteolytic process. In contrast to previous approaches we extended the model to incorporate endoproteolytic processes and present a method to construct a degradation graph from mass spectrometry time series data. Based on a degradation graph and the intensities extracted from the mass spectra it is possible to estimate reaction rates of the underlying processes. We further suggest a score to rate different degradation graphs in their ability to explain the observed data. This score is used in an iterative heuristic to improve the structure of the initially constructed degradation graph.

**Conclusion:**

We show that the proposed method is able to recover all degraded and generated peptides, the underlying reactions, and the reaction rates of proteolytic processes based on mass spectrometry time series data. We use simulated and real data to demonstrate that a given process can be reconstructed even in the presence of extensive noise, isobaric signals and false identifications. While the model is currently only validated on peptide data it is also applicable to proteins, as long as the necessary time series data can be produced.

## Introduction

Our view of proteases has changed drastically over the last two decades. Once thought to be only associated with simple protein degradation processes they are now recognized to play an important role in a variety of fundamental biological processes across species [1]–[3]. Furthermore, also their general importance in complex diseases such as cancer or HIV was described [4]–[8] and they were recognized as possible drug-targets [7], [9]. In the last decade, researchers also began to look more closely into the dynamics of proteolytic processes. It was found that changing dynamics of specific protease activity can be used to draw conclusions about an individual’s health condition [10], [11]. In fact, it was shown that the activity could also be used to distinguish different types of cancer [12].

Measuring and analyzing the dynamics of proteolytic processes often relies on array-based systems (see for example [13]), which have a high sensitivity. But this comes at the expense of high specificity to a single proteolytic process. In contrast to this we present a new method, that is able to reconstruct a proteolytic process and its kinetic parameters from mass spectrometry time series data. Mass spectrometry has become an essential tool in the field of proteomics [14] and can be used for the analysis of complex biochemical events, such as proteolytic processes (for a good overview see [15]).

**Figure 1:**
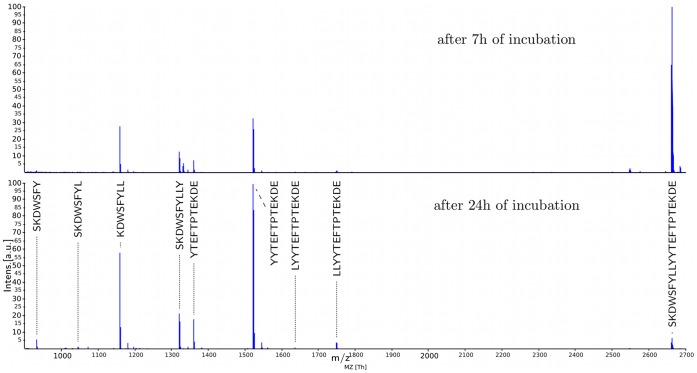
Degradation of a beta-2-microglobulin fragment observed via Mass Spectrometry. Mass spectra generated during the degradation of a beta-2-microglobulin fragment by a mixture of urine proteins after 7 (upper) and 24 (lower) hours of incubation. Intensity is given in percent of maximal peak intensity. In the lower spectrum all fragments were annotated that could be verified by MS/MS identifications. Details of data acquisition and sample preprocessing are given in the results section. All raw spectra of this time series are shown in the Supporting Information (Figure S2).

The basic idea in these experiments is to incubate peptides (or proteins) with one ore many proteases and to generate mass spectra in every chosen time step that reflect snapshots of the proteolytic process. Figure 1 shows two snapshot spectra of such an incubation experiment after seven and 24 hours of incubation. One can clearly see how a large peptide of about 2680 Da (represented by the large peak to the right in the upper spectrum) is degraded into smaller fragments (represented by large peaks to the left in the lower spectrum). The fragments (represented by peaks in the lower spectrum) are generated by two different degradation reactions: exo- and endoproteolytic cleavage. During an exoproteolytic reaction a single amino acid is removed from one of the free termini of a molecule, while in an endoproteolytic reaction the targeted molecule is cleaved at a position between the N- and C-terminus.

Compared to similar state-of-the art approaches by Yi et al. [16] and Kluge et al. [17] our contribution to the field is the inclusion of endoproteolytic degradation while using differential equations to model the full dynamics of the underlying process. In contrast to that the work of Yi et al. was only focused on a single proteolytic process (the degradation of fibrinopeptide A) and the method presented by Kluge et al. only considered exoproteolytic reactions and used a statistical model to describe the dynamics, independent of the degraded molecules.

**Figure 2:**
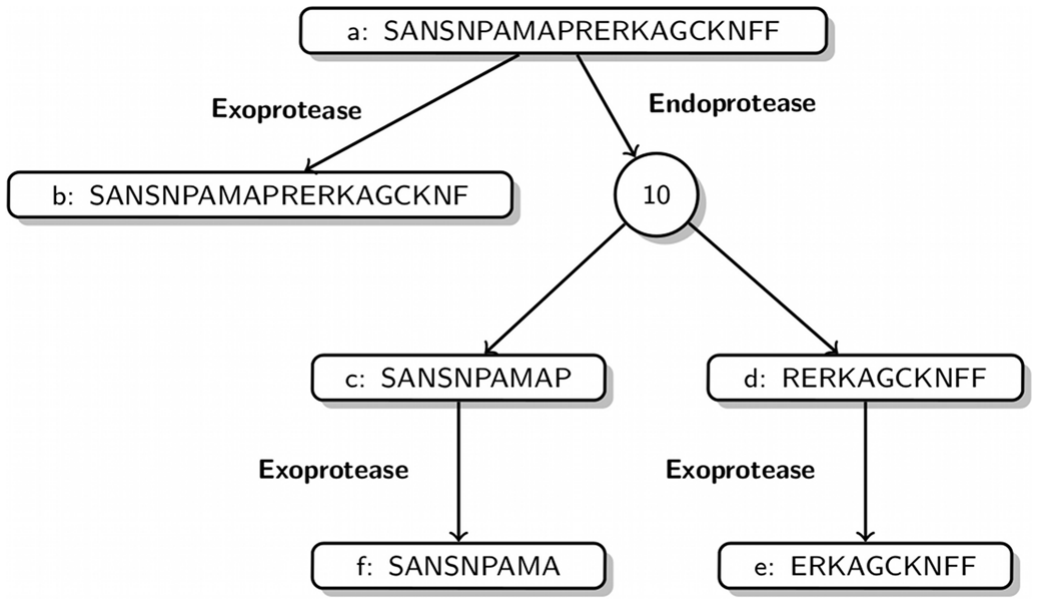
Complex proteolytic reaction visualized as graph. Example protease system acting on a single peptide (SANSNPAMAPRERKAGCKNFF) and the resulting degradation products. The shown reactions are all artificial.

An example result of our method is shown in Figure 2. Here we show a *degradation graph*, a data structure we will introduce later in detail, illustrating how a small peptide is degraded during several steps into smaller fragments. The kinetic constants of the individual reactions are omitted for the sake of clarity. The workflow of our method - which will be described in more detail in the remaining part of this paper - is as follows:

- Perform incubation experiment and generate mass spectra at every chosen time point.
- Create an initial degradation graph from the time series.
- Optimize the degradation graph structure by removing unlikely reactions and peptides and estimate the kinetic parameters of the generated model.

The results of our method are the sequences of all intermediate peptides, the proteolytic reactions that connect those peptides, as well as the dynamics of all proteolytic events.

In the result section we intensively test our approach on multiple simulated data sets with varying conditions. It shows a good performance in recovering the original structure as well as the underlying reaction rates. We further prove the applicability of our method to a real data set using a time series of a peptide incubated with an unknown mixture of urine proteins.

## Methods

Biochemical processes (such as proteolysis) can be described by ordinary differential equations (ODEs). This allows to simulate and analyze a process and thus to draw conclusions about its properties, such as steady-states or changes in concentration of its constituents over time. A simple example for such a system is Tyson’s cell cycle model [18]. To visualize these ODE systems oftentimes graphs are used, where nodes are the reactants and edges between them are the reactions. Note that both representations (ODE and graph) are equivalent. For modeling and visualizing proteolytic processes Kluge et al. introduced the *cleavage graph* [17] which they used to model exoproteolytic cleavage reactions. In the following we will extend this concept to also include endoproteolytic reactions. We call the resulting data structure *degradation graph* since it can be used to model all degradation reactions of a proteolytic process and also allows a convenient and comprehensible visualization.

### Degradation Graph

A proteolytic process where single or multiple peptides are generated by cutting peptides into smaller fragments can be modeled as a graph 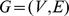.

The nodes *V* correspond to the degraded and generated peptides and the edges *E* to the proteolytic reactions. Since proteolysis is an irreversible reaction under physiological conditions the edges in the graph are directed from the degraded to the generated peptides.

As mentioned above, one can distinguish two types of proteolytic reactions, exoproteolytic reactions, where a single amino acid is removed from one of the free termini of the peptide, and endoproteolytic reactions, where the targeted peptide is cleaved at a position between the N- and C-terminus. For exoproteolytic reactions we connect two nodes with a directed edge from node *u* to *v* if we can obtain the amino acid sequence of *v* by subtracting a single amino acid from the beginning or the end of the amino acid sequence of *u*. For endoproteolytic reactions this is not that easy. Since we need to connect three nodes (the peptide that is targeted *u* and the two resulting fragments *v,w*) we need to break the idea of one reaction equals one edge in the graph. To ensure that we still associate the reaction with single edge, we introduce pseudo-nodes 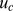, that represent the endoproteolytic process of cutting the peptide *u* at a specific position *c*. The pseudo-nodes can also be seen as representation of the endoprotease that cuts the peptide *u* at position *c*. We can now connect *u* to 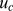 and associate all reaction specific information (e.g., reaction rate) with this single edge. We further connect 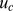 to *v* and *w* with so called pseudo-edges.

**Figure 3:**
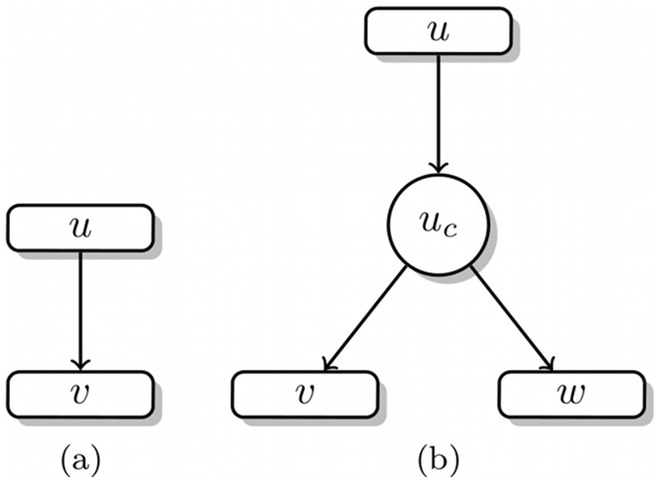
Representation of the basic degradation graph structures. (a) Exoprotease reaction, (b) Endoprotease reaction. See Figure 2 for an example containing both reaction types.

Both reaction types are separately shown in Figure 3. An example with real peptide sequences and both reaction types is shown in Figure 2.

### Constructing the Graph from Mass Spectrometry Data

In the previous section we defined the degradation graph and its relation to proteolytic processes. Now we present an approach to construct this graph based on series of *N* mass spectra collected at different time points 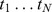 and a seed sequence *S* which we will also call base peptide from here on. Based on this input we try to identify signals in the mass spectra, that represent fragments of *S* produced by a proteolytic process. The seed sequence needs to be provided as input. It can for instance be the sequence of a known peptide probe that was incubated with an unknown mixture of proteases or a sequence taken from MS/MS identifications.

We shortly introduce some notation that eases the understanding of the following explanations. Given a node *v* in the degradation graph, 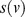 denotes the amino acid sequence of the peptide associated with the node *v*. The length of the amino acid sequence is given by 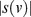. 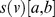 with 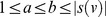 is the subsequence of the amino acid sequence from position *a* to position *b*. 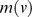 denotes the mass of the peptide associated with the node *v*. If we could identify a signal that corresponds to the peptide associated with *v*, we will denote it’s intensity with 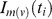. The association between mass and intensity takes into account, that mass spectrometers measure only mass to charge ratios and therefore cannot distinguish peptides with equal mass. Therefore different peptides with equal mass can be associated to the same intensity value, without counting the signal twice in the later analysis. The set of all peptide masses in the graph is denoted by *M*. We further introduce a queue of nodes *L*, which is empty at the beginning of the construction.

The construction of the graph is divided into two parts, verification and extension, which are executed on each of the input spectra. Before we can execute these steps, we need to initialize the degradation graph. This is done by adding a node for the seed sequence to the degradation graph. Afterwards we start with the verification step for the first spectrum recorded at time point 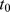, followed by the extension step. This is repeated for each of the input spectra. The pseudocode for both parts is shown in the Supporting Information (Figure S1).

**Figure 4:**
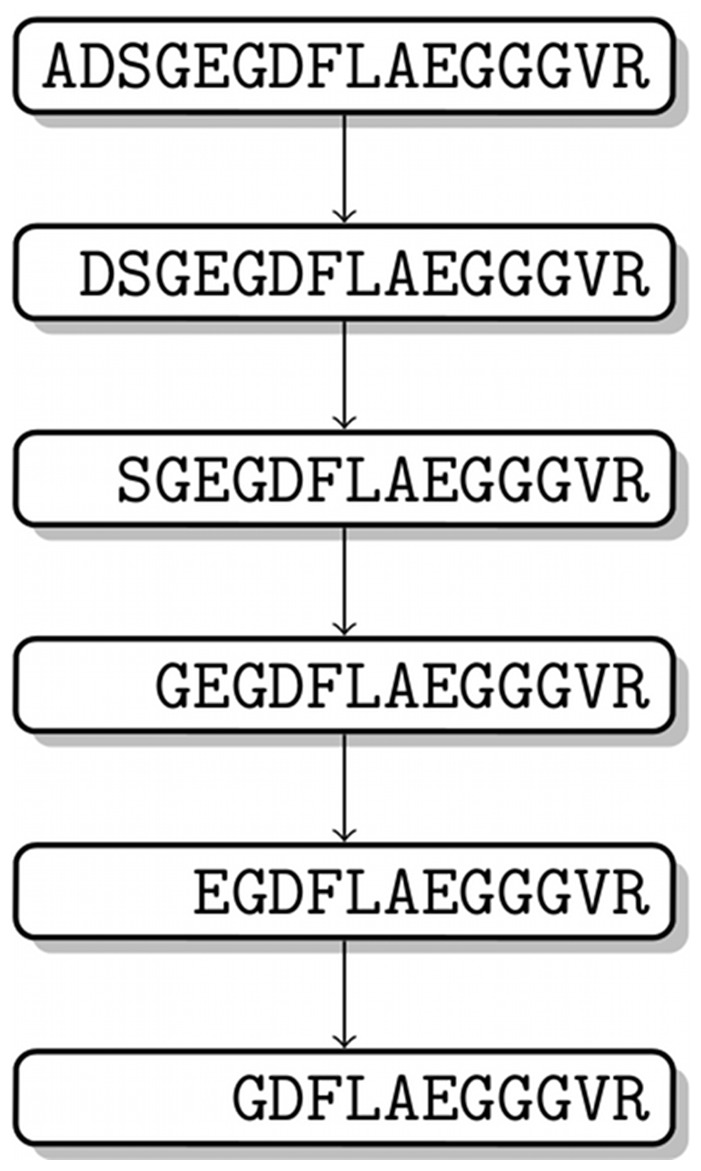
Degradation graph of the degradation of fibrinopeptide A (FPA) as reported in [**16**].

#### Verification

The first step is the verification of the degradation graph on the new spectrum. We therefore check for each node in the degradation graph whether we can find a signal that corresponds to this node in the spectrum. In general, we will identify signals by peptide mass fingerprinting [19]. Our approach is described in the Supporting Information (Text S1). Existing MS/MS identifications [20] are solely used for validation, since relying only MS/MS identifications during the construction phase of the algorithm would introduce a bias towards the used acquisition strategy. Each node *v* that could be identified in the spectrum is added to *L* and annotated with the observed intensity 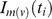.

#### Extension

The extension step is performed on the current spectrum as long as *L* is not empty. In each cycle a node *u* is removed from *L* and the following procedure is executed.

Given the node *u*, we start by removing the N- and C-terminal amino acid separately from 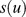 to simulate exoproteolytic degradation and search for the corresponding signals. If we find a signal we add the corresponding node *v* to the graph, annotate it with the signal intensity 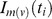, set it’s sequence 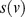 to either 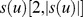 or 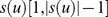, and connect the nodes *u* and *v* by an edge pointing from *u* to *v*. The generated node *v* is appended to the list *L*.

Subsequently we simulate the endoproteolytic reactions by splitting the sequence 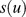 in two parts at each position *c* with 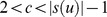. If we can identify both fragments of such a split in the mass spectrum, we add a pseudo-node 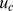, annotated with the sequence 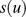 and the cutting position *c* to the graph and connect it to the degraded node *u*. We then add nodes *v* and *w* for each of the fragments to the graph, annotate it with the corresponding signal intensities (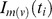, 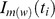), the sequences (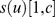 and 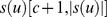), and connect it to the pseudo-node 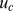. The generated nodes 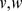 are appended to the list *L*.

### Estimation of Kinetic Parameters

After we generated the model representing the proteolytic process, i.e., the degradation graph, the next task is to estimate the kinetic parameters of the underlying process. To achieve this we first generate a system of ordinary differential equations (ODE) based on a degradation graph as described in the following section. For this system we estimate the kinetic parameters based on the observed signal intensities.

#### Generating an ODE Model for the degradation graph

Following the ideas presented by Yi et al. [16] the mathematical model is derived by the law of mass action and each proteolytic reaction is modeled as a first-order reaction, i.e., the rate of the reaction depends on the concentration of only one reactant. In case of proteolytic reactions, this reactant is the protein or peptide that is degraded. We neglect side effects like saturation of the degradation products but incorporating these would be possible by an extension of the ODE system. We write the rate equations for an exoprotease reaction, where *u* is degraded to *v* as follows
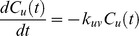


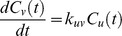
where 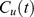 and 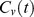 denote the concentration of peptide *u* and *v* at time *t*. 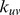 is the kinetic rate constant for the reaction. Endoprotease reactions are represented in the same manner with the slight difference that we need to model both degraded products.



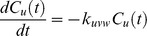


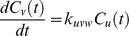


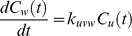



This transformation can be done for each reaction and each reactant in the degradation graph. As an example we transformed the degradation graph shown in Figure 2 into the following system of differential equations.
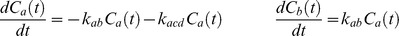


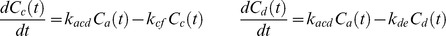


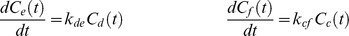



Since the degradation process as well as the mass spectrometry measurements happen *ex-vivo*, the base peptide (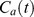 in the above example) has a fixed starting concentration and there will be no further production of the base peptide. In settings where this does not hold, one would need to explicitly model the generation of the base peptide into the equations (e.g., by a constant generation rate).

**Figure 5:**
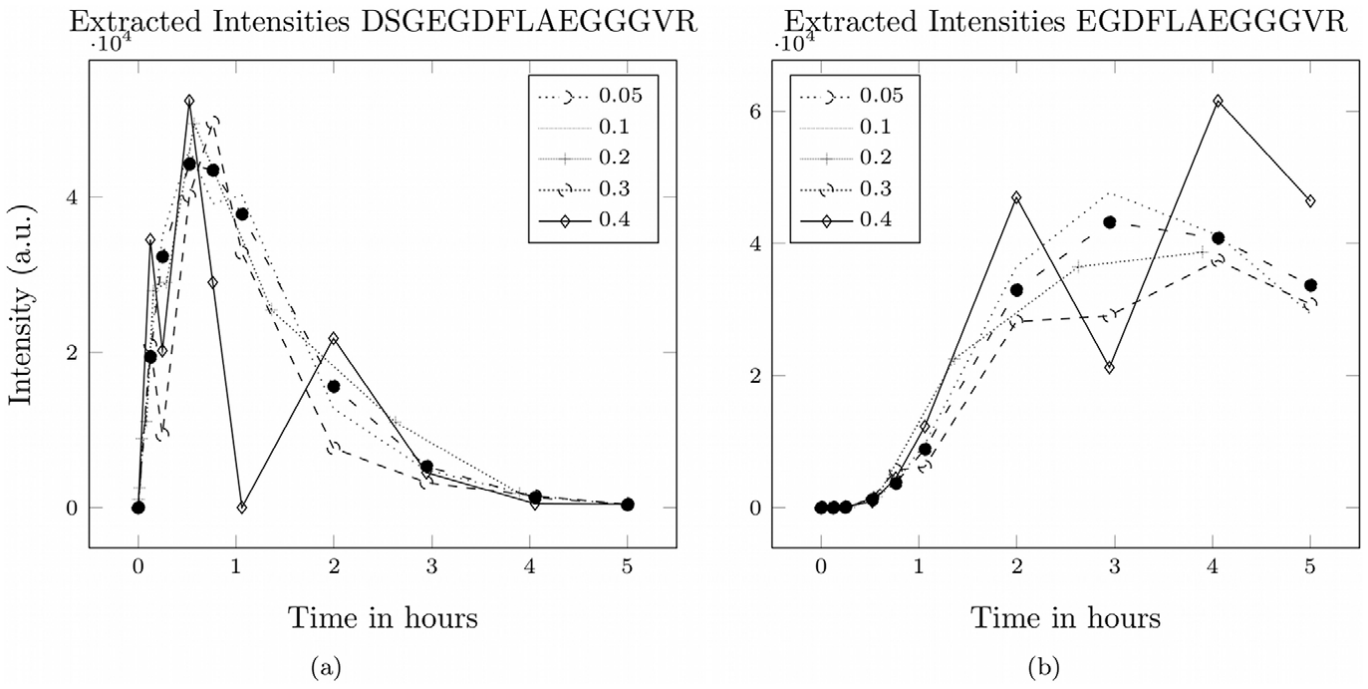
Effect of the different signal variability settings on the simulated signal intensities. Shown are the extracted signal intensities for two peptides (a) DSGEGDFLAEGGGVR (left) and (b) EGDFLAEGGGVR (right) of the fibrinopeptide A system shown in Figure 4 with increasing signal variability values.

**Figure 6:**
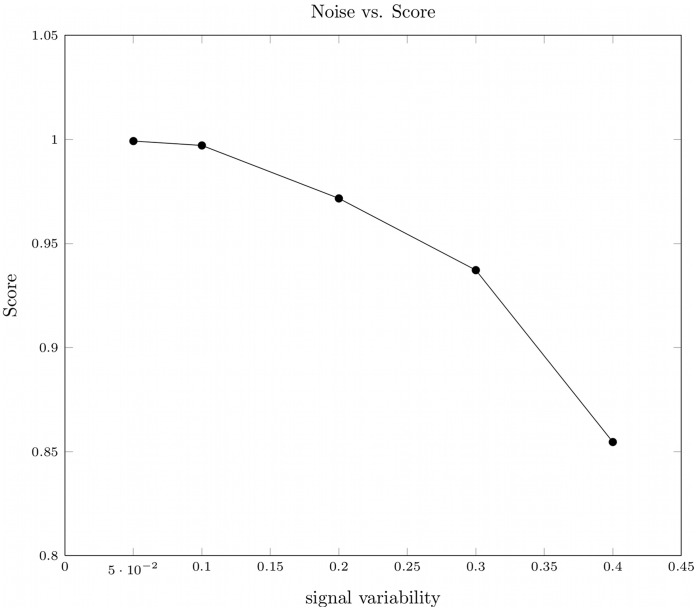
Effect of the signal variability on the score 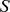. Effect of the variability of the signal with respect to the intensity on the score 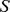 computed by our method. Data was generated based on the fibrinopeptide A system shown in Figure 4.

**Figure 7:**
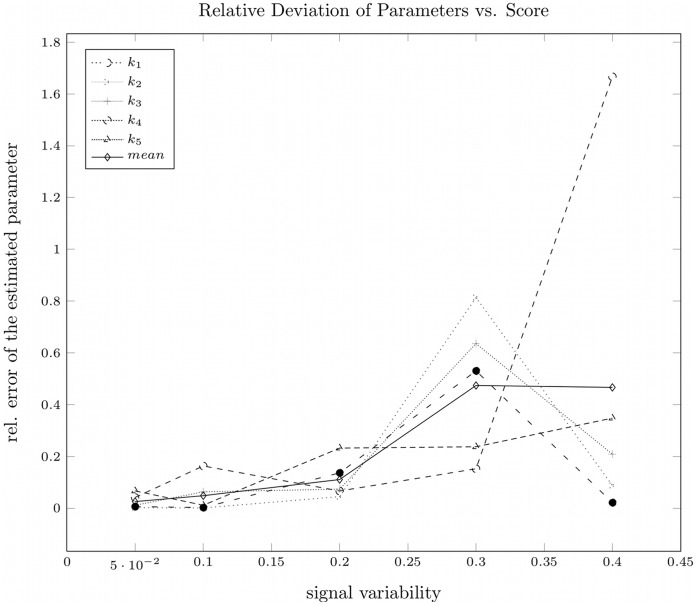
Effect of the signal variability on the the relative error of the estimated parameters. The quality is given in terms of the relative deviation of the estimated from the real parameter 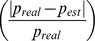. Data was generated based on the fibrinopeptide A system shown in Figure 4. The reaction parameters are numbered in the order of degradation (e.g., FPA 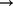 FPA-1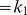) shown in Figure 4.

#### Transforming peptide concentrations to signal intensities

The presented ODE model is based on concentrations of peptides but with a mass spectrometer we can only observe intensities associated with a specific mass. The obvious question is what kind of relationship exists for a single peptide between its concentration and the intensity observed with the mass spectrometer. Moreover one cannot guarantee that two peptides with equal concentration will have the same intensity in the mass spectrometer.

Different studies [21], [22] have shown that for a single peptide a linear relationship between intensity and concentration is a reasonable assumption. Based on this we introduced a linear transformation from the model concentrations to the predicted signal intensities.
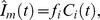
where 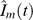 is the intensity associated with the mass *m* at time point *t*, *m* is the mass of the peptide 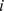, 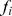 is a peptide specific factor, and 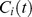 the concentration, computed by the model, for peptide 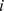 at time point 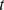. Yi et al. [16] already used a similar transformation successfully in their study. This transformation implicitly solves also the second problem of comparability between two observed intensities. Since each observed intensity will be transformed individually into the common concentration domain, the resulting concentrations can be compared afterwards. This transformation can also be used to compensate for systematic effects that occur in each measurement, e.g., quantification errors or incomplete ionization.

Another problem is that it can happen that two or more different peptides have the same or a nearly identical mass. These isobaric peptides cannot be distinguished in a mass spectrum. We therefore transform them into a single intensity value. For every observed mass 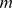, we compute a linear combination of all peptide concentrations, of peptides with a mass equal (or nearly equal) to 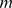.
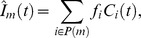
where 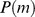 is the set of all peptides 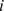 which have a mass of 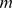.

#### Estimating reaction rates

To estimate kinetic parameters we first generated an ODE model based on a degradation graph as described above. We now need to find the optimal set of model parameters (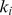) as well as transformation parameters (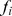), so that the difference between the computed model intensities 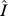 and the observed intensities 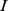 is minimal. Following standard practice we use a weighted sum of least squares differences between observed and model intensities as an error measure.
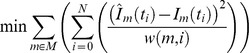
where 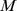 is the set of all observed masses, 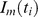 is the intensity observed for mass 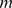 at time point 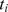, 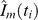 is intensity predicted by ODE system for the mass 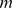 at time point 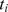, and 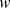 is a weighting function. The weighting function can for instance be used to use relative instead of absolute deviations, i.e.,



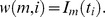



This is used to reduce the effect of different intensities being on different orders of magnitude. This minimization problem can theoretically be solved by any available optimization technique. After testing different available techniques we decided to use POEM, a Matlab-based version of BioPARKIN [23], [24], to estimate the model parameters as well as the transformation parameters. We further use POEM to estimate the initial concentration of the base peptide. POEM is based on damped Gauss-Newton techniques for solving the above optimization problem. Lack of robustness of damped Gauss-Newton techniques as observed often in model discrimination contexts, see [25], can be overcome by using dimension reduction in parameter space [26].

**Figure 8:**
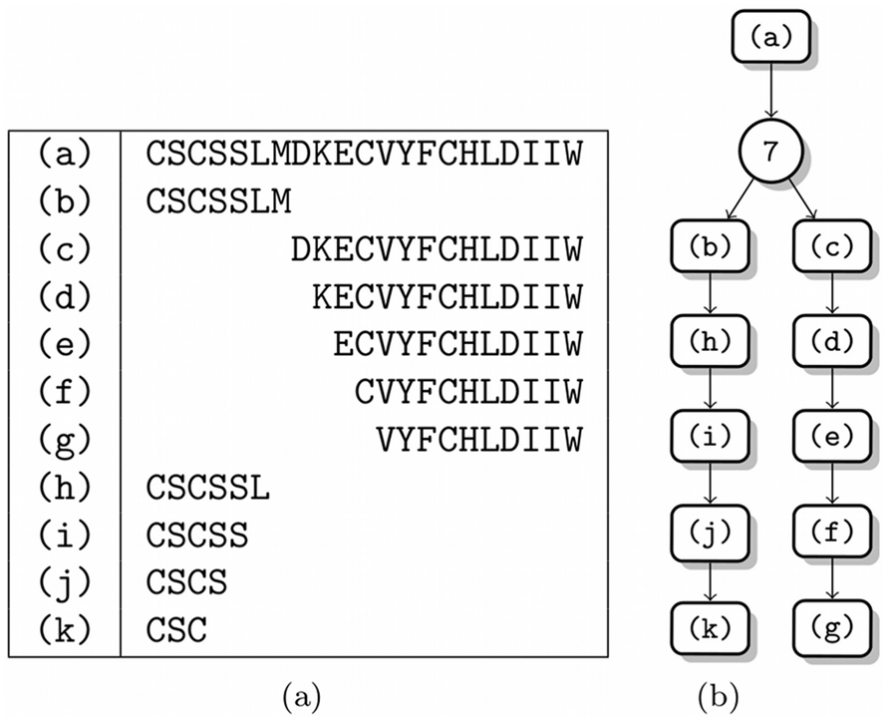
Endothelin-1 test system. Degradation of endothelin-1 by multiple artificial endo- and exoproteases. (a) The mapping of indices to sequences. (b) The degradation graph. package.

**Figure 9:**
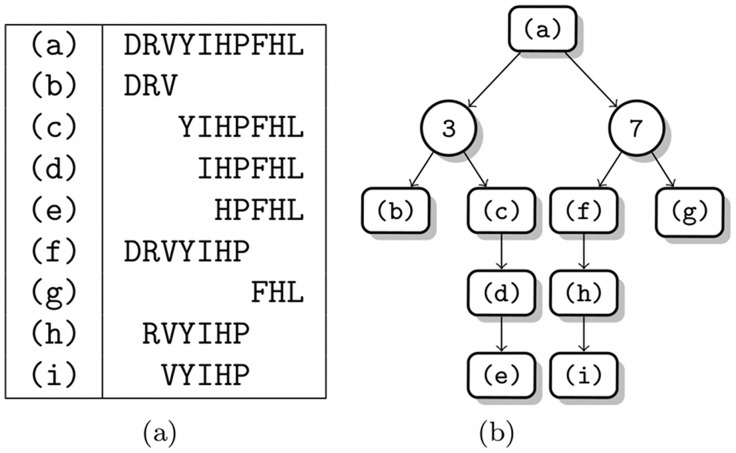
Angiotensin test system. Degradation of angiotensin by multiple artificial endo- and exoproteases. (a) The mapping of indices to sequences. (b) The degradation graph.

**Figure 10:**
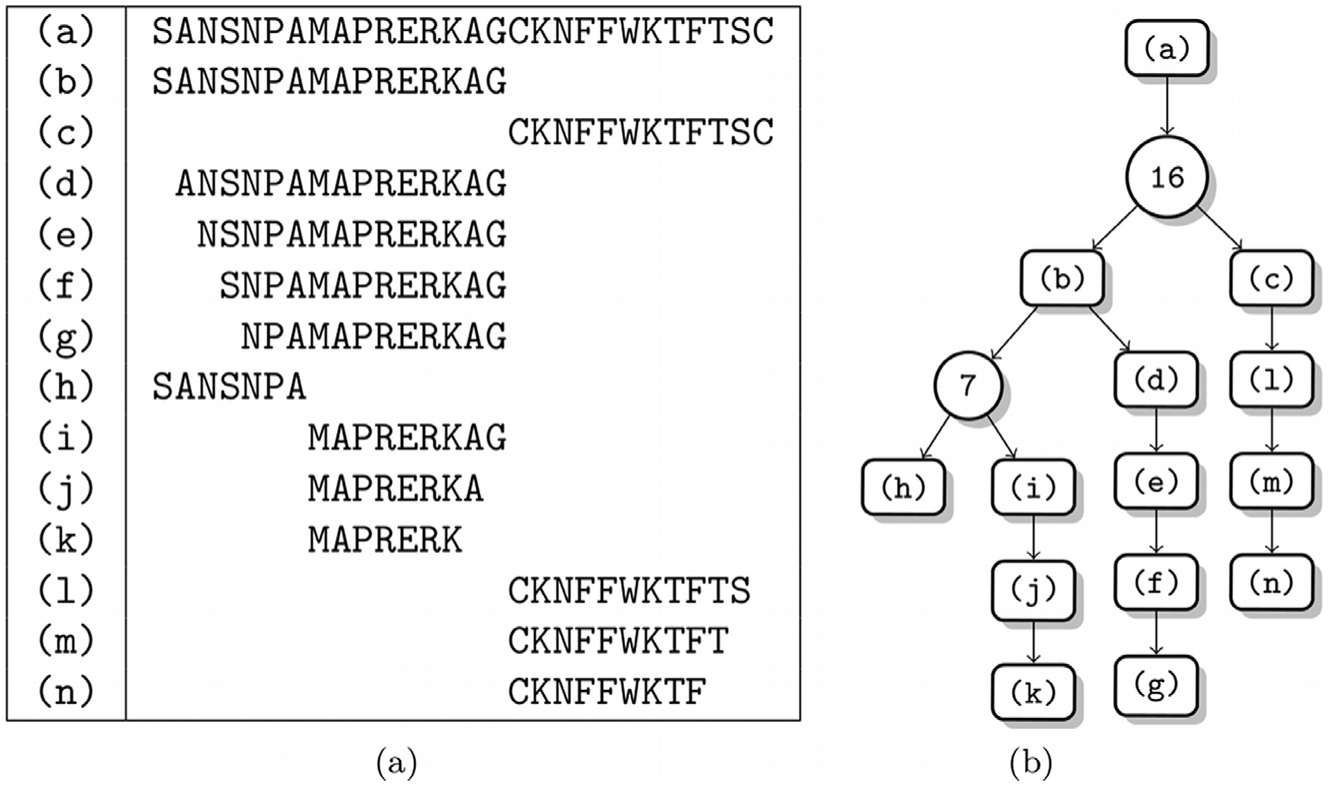
Somatostatin-28 test system. Degradation of somatostatin-28 by multiple artificial endo- and exoproteases. (a) The mapping of indices to sequences. (b) The degradation graph. package.

#### How to choose initial values

As the prior knowledge on the modeled system is very limited good initial values for the estimation of the model parameters are hard to find. We therefore chose the initial values based on the following scheme: For each node the edge (i.e., proteolytic reaction) is selected, which leads on the shortest path to the root node. For the corresponding reaction rate (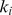) we assign an initial value of 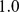. For all other incoming reactions the initial value is set to a value of 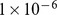. All transformation parameters (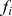) are set to 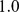.

### Evaluation and Optimization of the Degradation Graph Structure

The above presented approach to construct the degradation graph is greedy, i.e., it assumes that every signal in a spectrum that could match a subsequence of the base peptide is part of the proteolytic process and that every possible reaction occurred. This assumption is not always true. The signals could also originate from peptides with equal or at least similar masses as we have already seen in the previous section. But these peptides do not necessarily take part in the proteolytic reactions, that we want to model. We will call such peptides *decoy* peptides. Alternatively we may have multiple reactions to explain the formation of a peptide where only one is true. Hence, the degradation graph may contain peptides or reactions that did not occur in the actual underlying proteolytic process. To account for this we present a method to rank different subgraphs of an initial degradation graph with respect to their ability to explain observed data. Followed by a heuristic approach to construct a series of smaller models from the initially generated degradation graph without the need to compute every possible subgraph.

**Table 1:** Parameter estimation error for the endothelin 1 system.

Parameter	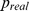	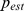	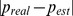	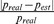
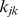	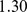	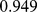	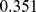	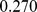
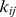	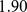	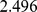	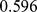	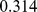
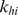	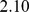	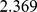	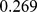	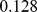
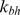	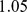	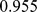	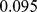	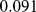
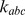	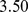	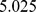	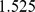	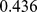
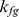	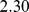	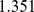	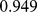	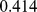
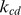	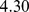	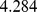	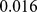	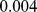
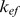	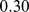	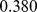	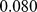	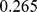
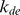	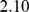	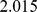	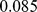	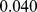

Relative and absolute deviations of the estimated parameter values for the endothelin 1 system. The indices for the parameter names are taken from Figure 8. 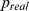 denotes the parameter values used for the initial simulation and 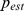 the value estimated by the presented approach. The last two columns contain the absolute and the relative deviation of the estimated from the real parameter value.

**Table 2:** Parameter estimation error for the angiotensin system.

Parameter	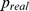	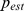	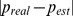	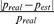
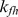	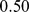	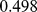	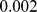	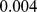
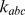	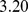	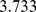	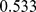	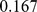
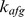	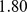	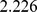	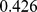	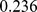
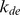	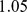	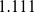	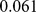	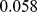
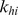	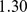	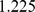	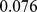	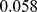
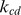	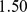	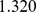	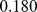	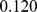

Relative and absolute deviations of the estimated parameter values for the angiotensin system. The indices for the parameter names are taken from Figure 9. 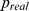 denotes the parameter values used for the initial simulation and 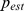 the value estimated by the presented approach. The last two columns contain the absolute and the relative deviation of the estimated from the real parameter value.

**Table 3:** Parameter estimation error for the somatostatin 28 system.

Parameter	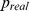	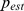	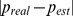	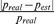
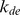	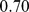	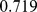	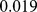	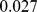
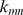	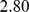	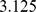	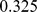	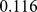
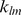	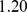	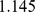	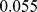	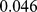
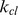	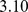	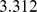	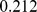	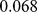
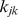	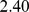	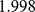	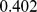	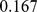
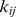	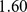	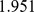	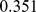	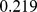
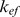	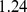	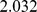	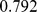	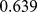
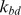	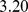	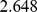	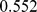	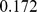
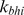	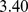	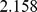	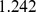	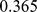
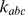	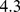	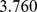	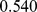	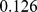
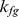	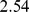	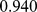	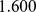	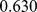

Relative and absolute deviations of the estimated parameter values for the somatostatin 28 system. The indices for the parameter names are taken from Figure 10. 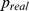 denotes the parameter values used for the initial simulation and 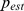 the value estimated by the presented approach. The last two columns contain the absolute and the relative deviation of the estimated from the real parameter value.

#### Evaluating different models

To find the degradation graph that optimally explains the observed data it is necessary to rank the different graphs. Here we describe a scoring scheme that can be used to rank the generated models.

To ease the following explanations we will introduce some further notation. Given a degradation graph 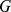, a subgraph 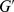 is defined as 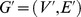, where 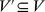 and 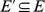. We also require that 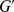 is connected, i.e., for all pairs of nodes 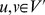 exists a path of length 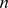 in 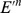 that connects 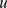 and 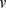. The subgraph 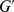 also defines 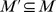 as the subset of all masses 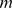 and their associated intensities that are explained by the subgraph 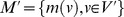.

The proposed score consists of two components. The first score component 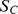 is the average Pearson correlation of the intensities predicted by the model (with estimated reaction parameters) and the actual observed data. This component should reflect the goodness of fit between the measured intensities and the computed model intensities. We compute for each explained mass 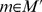 the Pearson correlation 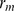 between the observed intensity values and the predicted values from the model.



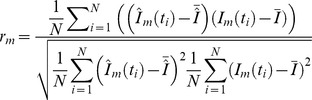
.
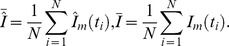



We then use the mean of all Pearson correlation values as measure for the goodness of fit.
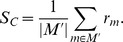



The second component of the score 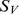 is the part of the standard deviation of the original degradation graph, that is conserved by the specific subgraph.
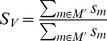
where 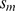 is the standard deviation of the signal corresponding to the mass 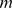. 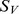 reflects the ability of the subgraph to explain the important parts of the originally collected signals.

To compute a single score 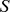 from these two components we build the weighted sum of both scores.
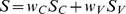



To determine good weights 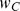 and 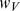 we carried out several experiments on simulated data. A weight of 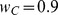 for the correlation score 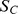 and 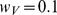 for the variability score 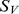 showed the best separation of the correctly and wrongly identified models. For datasets with low quality (e.g., due to high amounts of noise or too few sampling points) weights of 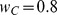 and 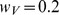 have shown a good performance. For such datasets we expect a less reliable fit for the time series and therefore decreased the weighting factor for the quality of the fit.

**Figure 11:**
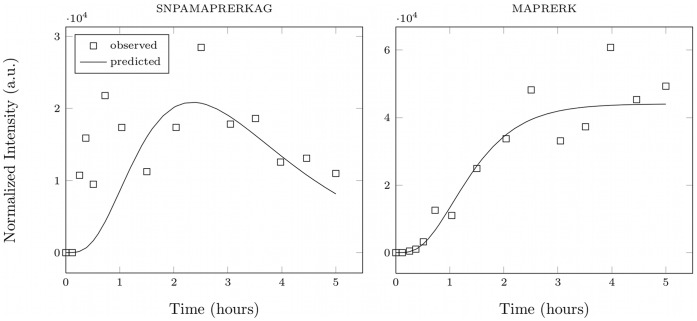
Intensity course for different fragments of the somatostatin-28 test system. Shown is the intensity course of two peptide fragments compared with the predicted model intensities for the best somatostatin-28 degradation graph.

**Figure 12:**
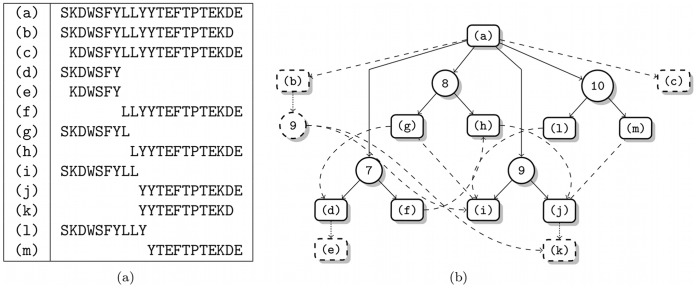
Initial degradation graph for the beta-2-microglobulin fragment estimated from real data. Shown is the degradation graph for the beta-2-microglobulin fragment which was initially estimated from a MALDI time series. (a) The mapping of indices to sequences. (b) The initial degradation graph. The dashed edges and nodes represent those reactions, that were not validated manually.

**Figure 13:**
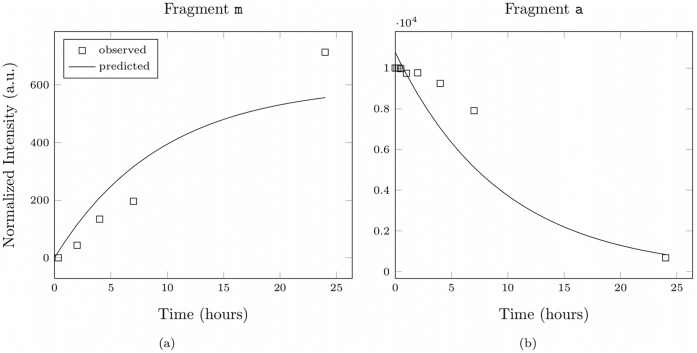
Intensity course for different fragments of the manually validated degradation graph. Intensity course for different fragments of the manually validated degradation graph. See text for more details.

#### Heuristic search for the optimal graph

Constructing all possible subgraphs, generate the associated ODE system, and estimating the corresponding reaction and transformation parameters is possible for small graphs. With increasing size in terms of number of nodes and reactions, estimating the reaction and transformation parameters for all subgraphs gets more computationally intensive. If we want to generate each possible combination of reactions we would get 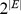 possible subgraphs. Even if we filter out some of the subgraphs (e.g., those who do not contain the root node or are not connected) we would still have to consider exponentially many subgraphs. For each of these subgraphs we would then need to derive the associated ODE system and estimate the reaction and transition parameters.

To speed up this procedure we present a heuristic approach. Preliminary tests have shown that the presented graph score improves, if the structure of the degradation graph gets closer to the original one. This can be explained based on the composition of the score. The first component reflects the goodness of fit between model and observed data. This should improve if we remove peptides and reactions, that do not belong to underlying process. The second component reflects the variability of the signals. If we remove only nodes that do not participate in the reaction, i.e., whose variability is low compared to the signals of peptides which are degraded and produced, this score component should still be near to the optimal value.

Based on the construction algorithm we know that the identified degradation graph is maximal in the sense that it contains all signals that were produced by the assumed process and possibly also parts that do not belong to the process. To find the optimal subgraph we start by removing all terminal reactions of the graph (i.e., reactions that produce at least one leaf) separately. For each of these subgraphs we estimate the kinetic parameters as described earlier. Subsequently we rate all subgraphs according to the criteria presented above. Then we take the best 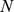 models and again remove all leafs separately. We continue with this procedure as long as we can find at least one graph whose score is under the top 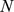 of all so far computed subgraphs and that was not trimmed in a previous iteration.

With this approach we can drastically reduce the amount of parameter optimizations that need to be carried out by still finding the originally embedded graph.

Preliminary tests have shown that setting 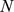 to either 2 or 3 is sufficient to effectively bound the number of unnecessary model evaluations while still identifying the original degradation graph.

### Run-time Considerations

The above presented combination of degradation graph construction, parameter estimation and structure optimization requires a considerable amount of time, if the initial degradation graph is large. Therefore we now describe an approximation of the running time in the worst case. The run time of the initial degradation graph construction is determined by the number of verifications needed. Under the assumption that we would construct the complete degradation graph, i.e., all peptides are degraded in every possible way, one would create a degradation graph, which contains all possible substrings of the initial peptide sequence. Since we would need to verify each of this substrings once, the running time is in the worst case bounded by the maximal number of possible substrings of the initial peptide sequence. Given a seed sequence of length 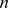 we can construct at most 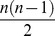 possible fragments, which could be checked in the spectrum. If we now analyze 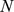 spectra we will have at most 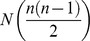 verifications.

The complexity of the parameter estimation procedure can be approximated by 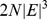, where 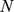 is the number of time points, i.e., the number of evaluated mass spectra, and 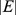 is the number of unknown parameters, i.e., the number of edges in the graph minus the number of edges connecting pseudo- and real nodes. Given this the time required for the parameter estimation will decrease with the subgraphs getting smaller. Under the assumption that even the proposed heuristic could require the computation of each subgraph, we would need to trigger 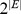 optimizations in the worst case.

## Results

To evaluate the ability of the presented approach we have to consider two parts: (1) reconstruction of the correct sequence of proteolytic events and (2) estimation of the reaction rates. The influence of different parameters like the complexity of the degradation graph or the variations of the signals in the mass spectrometer have to be assessed. This can only be done if enough data is available in terms of number of samples in varying quality. Both is not always given.

To overcome this problem we designed a series of simulated mass spectrometry data sets. The mass spectra were simulated using the software MSSimulator [27], a comprehensive simulator for mass spectrometry data. A detailed description of the software is given by Bielow et al. [27]. MSSimulator generates mass spectra based on a set of amino acid sequences and a configuration file, which contains all parameters necessary for the simulation, like ionization type or instrument resolution. In the following experiments all configuration parameters are hold fix, expect the signal variability. The signal variability is an intensity dependent deviation of the signal intensity of a single peptide signal, i.e., if we set an intensity noise value of 10% of the total signal intensity (area under the curve of the simulated peak) will vary with a standard deviation of 10% of the original signal intensity.

The time series for the simulated proteolytic process is generated based on the associated ODE system. The produced peptide concentrations are combined with the peptide sequences and are then put into MSSimulator.

All input and configuration files can be found in the Supporting Information (File S1, File S2). All generated mass spectra are post-processed by the OpenMS PeakPicker [28] to transform the raw spectra into manageable pairs of mass-to-charge ratio and intensity.

We evaluate our approach on four different simulated models and one real data set. The first one is the degradation of fibrinopeptide A presented in [16], which is used to show the general performance under varying noise conditions. The later three are artificial systems constructed to show the applicability of the method to complex proteolytic processes. The real data set is a series of mass spectra collected during the incubation of a peptide probe with urine proteins.

### Study 1 (Simulated Data): Validation Using the *ex vivo* Degradation of Fibrinopeptide A (FPA)

To demonstrate that our approach is able to recover the correct sequence of proteolytic events, i.e., the degradation graph and the corresponding kinetic parameters, we simulated a data set based on the fibrinopeptide A (FPA) (Swiss-Prot:P02671[20–35]) degradation, as described in [16]. It consists of a series of exoproteolytic cuts at the N-terminus of FPA. The corresponding degradation graph is shown in Figure 4. For the proteolytic reactions we used a slightly modified version of the kinetic parameters as published in [16]. The modified parameters can be found in the ODE formulation of the Supporting Information (File S1).

The proteolytic system was simulated over a time of 5 *h*. We generated 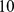 sampling points for the time series, five during the first hour of the incubation and the other five distributed equally over the remaining 4 hours. For these 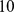 time points we generated five sets of mass spectra with increasing signal variability of 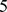, 10, 20, 30, and 40% of the original signal intensity. The impact of the signal variability on the time course of the peptide intensities is shown in Figure 5. Thereafter we applied our new method to estimate the model structure as well as the kinetic parameters for each of the five time series.

Our method succeeded to reconstruct the original degradation graph as it is shown in Figure 4. The scores computed for the reconstructed systems show a clear dependency on the noise added during the mass spectra simulation (see Figure 6). The relative error for the individual parameters of the system in relation to noise on the simulated data is shown in Figure 7. These experiments show that even in the presence of extensive noise a valid reconstruction of the original process is possible. Also the estimated parameter values have an acceptable agreement with the original parameters. With a signal variability of 30% the quality of the estimated parameters starts to decrease drastically. This could possibly be mitigated by increasing the number of sampling points.

### Study 2 (Simulated Data): Complex Degradation of Human Plasma Peptides

To test our method in a complex setting where also endoproteolytic reactions occur, we simulated the degradation of several human plasma peptides (and peptide fragments) by multiple artificial endo- and exoproteases. The targeted peptides were fragments of endothelin 1 (Swiss-Prot:P05305[53–73]), angiotensin (Swiss-Prot:P01019[34–43]), and somatostatin-28 (Swiss-Prot:P61278[89–116]). The full set of reactions and the corresponding peptide sequences are shown in Figures 8, 9, and 10.

All three systems were again simulated over a time of 5 *h*. We generated 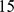 sampling points from the time series. More sampling points were generated in the first hour of each time series, since during this time the systems change most. For all time points we generated mass spectra with a signal variability of 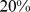. During the mass spectrometry simulation of the systems we added decoy peptides that have masses similar to possible fragments of the base peptides. Therefore we also applied our method to iteratively optimize the structure of the degradation graph.

Our method generally succeeded to reconstruct the originally simulated degradation graphs. In case of the angiotensin system the peptide (e) was misinterpreted as IHPFH. Since both terminal amino acids of its predecessor (Leucin and Isoleucin) have equal mass they cannot be distinguished by the mass spectrometer hence both solutions are equally good.

For all three systems the estimated parameters in comparison to the original parameters are shown in Tables 1, 2, and 3. In general, the recovered parameters are quite well estimated. The average relative deviation between the estimated and the real parameters is between 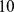 and 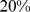 for the different experiments. It can be observed that the largest errors occur towards the end of the degradation process (e.g., 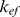 for the somatostatin 28 system). This can be due to the late formation of the later products and with this the lack of enough data points to effectively estimate the reaction parameters. An extension of the sampling range beyond 5 *h* or an increased sampling rate could possibly solve this issue.

Figure 11 shows the extracted intensities of two characteristic somatostatin-28 fragments compared with predicted model intensities. As one can see the predicted model intensities and the simulated intensities show a good agreement in their dynamic behavior.

### Study 3 (Real Data): Validation on MALDI Time Series Data

To demonstrate the applicability of our method to experimental data, we analyzed a data set where a fragment of beta-2-microglobulin (Swiss-Prot:P61769[77–97]) was incubated with different urine proteins. Manual inspection of the mass spectra combined with the analysis of MS/MS spectra lead to the assumption, that four endoproteolytic reactions at positions 7–10 occurred. We applied the presented method to the dataset to validate this assumption.

The mass spectra for two time points (*t* = 7 h and *t* = 24 h) are shown in Figure 1. In the mass spectrum for *t* = 24 h the peaks for the fragments generated by the four endoproteolytic reactions as well as the base peptide are annotated. A figure showing all mass spectra is included in the Supporting Information (Figure S2).

#### Data acquisition and preprocessing

For the immobilization of urine proteins from haemolytic urine of renal transplantation patients CNBr-activated Sepharosebeads® 6 MB were used. The Sepharosebeads® were incubated in 0.1 M hydrochloric acid (HCl) on a mixer (Horizontal Shaker, Rotator Drive STR4 Stuart Scientific, Redhill, England) for 30 min and washed with HPLC-grade water. The immobilization of urine proteins onto the Sepharosebeads® was done in coupling-buffer (100 mM 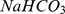, 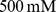 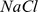, pH 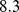) during an incubation period of 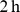 on a mixer. Per preparation 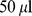 urine and 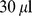 Sepharosebeads® were used. After immobilization the Sepharosebeads® were washed with HPCL-grade water. Free binding capacities were saturated by over night incubation at 4°C in blocking-buffer (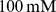 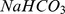, 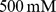 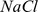, 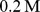 Glycin, pH 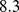). Afterwards the blocking-buffer was removed by washing with HPLC-grade water repeatedly.

Incubation of immobilized urine proteins took place in sodium acetate buffer at pH 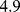 and was started by addition of the beta-2-microglobulin fragment to the immobilized proteins with a final concentration of 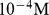 in a reaction volume of 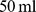. At nine distinct time points aliquots were taken from the reaction mixture and diluted in a ratio of 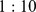 in 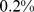 (v/v) formic acid (Fluka/Sigma-Aldrich, Steinheim, Germany) for MALDI-TOF/TOF analysis on a 4700 Proteomics Analyzer (Applied Biosystems). The distinct time points were after 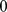, 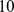, 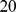, and 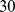 minutes, and 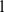, 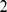, 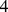, 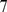, and 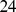 hours.

All mass spectra were preprocessed as described in the simulation studies. To account for the variability of the overall intensity between different mass spectra we applied a customized normalization strategy to the intensities of the collected signals, which is described in detail in the Supporting Information (Text S2).

### Results

The preprocessed spectra were analyzed by our method to identify the optimal degradation graph for the given mass spectra. The initially constructed degradation graph contains all four manually confirmed endoproteolytic cuts as well as four additionally not manually annotated exoproteolytic and one additional endoproteolytic cut. The complete degradation graph is shown in Figure 12. The unvalidated proteolytic reactions and fragments are represented as dashed nodes and lines.

It can further be seen from Figure 12 that the fragments generated by the validated endoproteolytic cuts are interconnected by exoproteolytic reactions. Although these reactions are possible, they are very unlikely and hence should be removed during the optimization. To reflect this the previously described selection of initial values was applied. Due to the lack of sampling points for the actual reactions, which took place between the last two sampling points, we have chosen the low quality weighting factors for this analysis (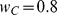 and 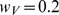).

Optimizing the degradation graph structure results in a list of subgraphs ranked by their scores. The scores varied widely with the different generated structures. A figure showing the development of the score is included in the Supporting Information (Figure S3). Since the correct solution is unknown, we need to inspect the list and the different proposed solutions. As expected, based on the manual validation (see above), the degradation graph with the highest score contains the four endoproteolytic cuts at positions 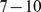. The unvalidated side reactions (see the dashed nodes and edges in Figure 12) were mostly removed, just two exoproteolytic reactions (fragment i to g and g to d) are still included, but have an estimated reaction rate of 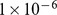. Although these reactions are still included in the degradation graph they have effectively no influence on the system and thereby can be neglected.

Figure 13 shows the observed and the predicted intensities for a subset of the peptide fragments of the degradation graph with the best score. The time courses of all peptides are shown in the Supporting Information (Figure S4). It can be seen that the measured intensities not always agree with the predicted intensity course, but they seem to show a comparable behavior. More time points especially in the time from 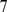 to 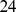 hours and an improved quantification (e.g., via a spiked in control sample) could further improve the results.

### Conclusion

In this paper we presented a new method to model any proteolytic process as a degradation graph including an algorithm to construct the degradation graph based on mass spectrometry time series data. The degradation graph can easily be translated into a system of ordinary differential equations, which can be used to estimate the kinetic parameters of the proteolytic process. We further proposed an approach to optimize the initially constructed graph in the presence of decoy and overlapping signals. It is based on a score, that is used to rank the optimized and the original degradation graphs in their ability to explain the actually observed data. Using simulated data we have shown that our approach is able to compute good estimates for the kinetic parameters of the ODE systems even in the presence of noise and decoy signals. With a careful preparation of the samples using accepted standard operating procedures [29] the variability of the mass spectrometry data is below the observed boundary of the presented method. Applied to real data our approach reconstructed manually validated endoproteolytic reactions and removed unvalidated reactions and peptides from the graph.

We are aware of other biochemical approaches that give a much more robust and exact estimate for the reaction rates, but most of these methods rely on a much more time consuming measurement of the reactants and their concentration and, more importantly, often require prior knowledge of all reactants, which is not necessary for our method.

Applications for this method can be to identify and characterize unknown proteases and the estimated reaction kinetics can possibly be used to classify between different sample categories as it was done in [17]. With the ability to handle also false identifications the method can even be used in complex samples.

Future directions are an extensive validation of the proposed approach on real data. Another by now unsolved issue is the handling of unobserved peptides, i.e., peptides that participate in the reactions, but are not observable in the mass spectra. This can be due to different reasons e.g., the peptide cannot be ionized by the mass spectrometer or the degradation process is so fast, that the generated peptide is degraded before it can be measured. This problem can be handled by a modification of the construction algorithm for the degradation graph, as long as a downstream peptide is again observable. Also the handling of more than one seed sequence would be favorable. Finally a robust integration of MS/MS identifications into the method could further improve its performance. This could be done in two ways: One could use MS/MS identifications during the initial construction of the degradation graph in combination with the already used PMF approach, as an additional and more reliable way to identify possible fragments of the peptide probe. Furthermore one could integrate the MS/MS identification and its score into the scoring function by penalizing the removal of highly scored identifications.

The proposed score could also be improved in future development. The approach would benefit from a score that does not require specific scaling parameters for the different components. It would remove the step of optimizing the scaling parameters. First experiments using a scaled least-squares residual were carried out on simulated data sets. Those have shown promising but not yet comparable results.

The presented method is available on request. The whole approach is integrated into the proteomics.net platform [30]. The estimation procedure requires POEM which is available, for academic use, on request from the Computational Systems Biology Group, Konrad-Zuse-Zentrum für Informationstechnik Berlin (ZIB) (http://www.zib.de/en/numerik/csb.html).

## Supporting Information

Figure S1 Pseudocode for the degradation graph construction.
(PDF)

Figure S2 All mass spectra of the beta-2-microglobulin time series.
(PDF)

Figure S3 Ranked subgraphs of the beta-2-microglobulin analysis.
(PDF)

Figure S4 Time Courses of the confirmed beta-2-microglobulin fragments and the predicted dynamics.
(PDF)

Text S1 A simple peptide-mass-fingerprinting (PMF) strategy to extract intensity values from mass spectra.
(PDF)

Text S2 Description of the normalization method used for the beta-2-microglobulin data set.
(PDF)

File S1 Matlab code to generate the input data for MSSimulator to simulate the artificial models.
(ZIP)

File S2 Configuration files for the post-processing of the simulated and real data sets.
(ZIP)
