# Supplementary material for: Inferring Proteolytic Processes from Mass Spectrometry Time Series Data Using Degradation Graphs
Source: PLoS One. 2012 Jul 17;7(7):e40656. doi: 10.1371/journal.pone.0040656 (PMC3398944; doi:10.1371/journal.pone.0040656)
Supplement: Figure S1 — Pseudocode for the degradation graph construction. (PDF) [file pone.0040656.s001.pdf]

Figure S1: Pseudo-code for the *degradation graph* construction

Pseudo code for the *degradation graph* construction. The notation was already defined in the text but for completeness and an easier reading we repeat it here.

Given a node v in the *degradation graph*, $s(v)$ denotes the amino acid sequence of the peptide associated with the node v. The length of the amino acid sequence is given by $|s(v)|$. $s(v)[a,b]$ with $1 \leq a \leq b \leq |s(v)|$ is the subsequence of the amino acid sequence from position a to position b. $m(v)$ denotes the mass of the peptide associated with the node v. If we could identify a signal that corresponds to the peptide associated with v, we will denote it's intensity with $I_{m(v)}(t_i)$. The association between mass and intensity takes into account, that mass spectrometers measure only mass to charge ratios and therefore cannot distinguish peptides with equal mass. Therefore different peptides with equal mass can be associated to the same intensity value, without counting the signal twice in the later analysis. The set of all peptide masses in the graph is denoted by M. We further introduce a queue of nodes L, which is empty at the beginning of the construction.

```
function VERIFICATION(Degradation Graph g, Spectrum P, Time t)
    L ← {}
    for each node v in g do
        if P contains a signal p for peptide s(v) then
            L ← {L, v}
            I_m(v)(t) ← intensity of p
        end if
    end for
    return L
end function
```

function EXTENSION(*Degradation Graph* g, *Spectrum* P, *Time* t, *Node List* L)
  for each node u in L **do**
    if P contains a signal p for peptide $s(u)[2, |s(u)|]$ **then**
      create node v, with $s(v) \leftarrow s(u)[2, |s(u)|]$ and $I_{m(v)}(t) \leftarrow$ intensity of p
      add edge $u \rightarrow v$
      $L \leftarrow \{L, v\}$
    end if
    if P contains a signal p for peptide $s(u)[1, |s(u)| - 1]$ **then**
      create node v, with $s(v) \leftarrow s(u)[1, |s(u)| - 1]$ and $I_{m(v)}(t) \leftarrow$ intensity of p
      add edge $u \rightarrow v$
      $L \leftarrow \{L, v\}$
    end if
    for each c, $2 < c < |s(v)| - 1$ **do**
      if P contains signals p_v, p_w for peptides $s(u)[1, c]$ and $s(u)[c+1, |s(u)| - 1]$ **then**
        create nodes u_c
        add edge $u \rightarrow u_c$
        create node v, with $s(v) \leftarrow s(u)[1, c]$ and $I_{m(v)}(t) \leftarrow$ intensity of p_v
        create node w, with $s(w) \leftarrow s(u)[c+1, |s(u)|]$ and $I_{m(w)}(t) \leftarrow$ intensity of p_w
        add edge $u_c \rightarrow v$
        add edge $u_c \rightarrow w$
        $L \leftarrow \{L, v, w\}$
      end if
    end for
  end for
end function
