# Supplementary figures and images for: Inferring Proteolytic Processes from Mass Spectrometry Time Series Data Using Degradation Graphs
Source: PLoS One. 2012 Jul 17;7(7):e40656. doi: 10.1371/journal.pone.0040656 (PMC3398944; doi:10.1371/journal.pone.0040656)

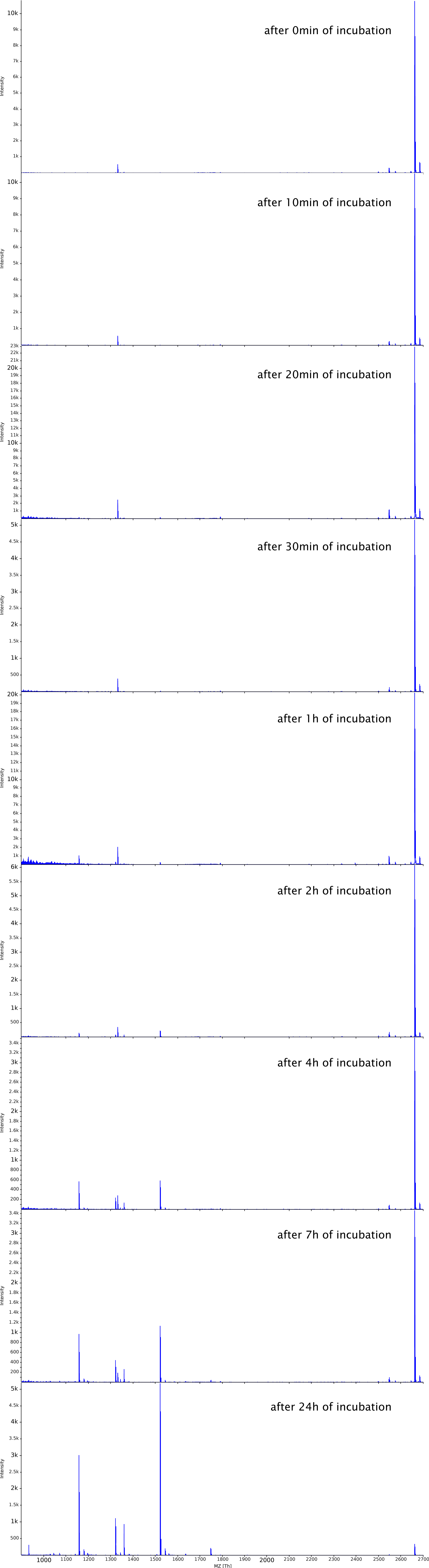

Supplement: Figure S2 — All mass spectra of the beta-2-microglobulin time series. (PDF) [file pone.0040656.s002.pdf]
