# Supplementary material for: Inferring Proteolytic Processes from Mass Spectrometry Time Series Data Using Degradation Graphs
Source: PLoS One. 2012 Jul 17;7(7):e40656. doi: 10.1371/journal.pone.0040656 (PMC3398944; doi:10.1371/journal.pone.0040656)
Supplement: Figure S3 — Ranked subgraphs of the beta-2-microglobulin analysis. (PDF) [file pone.0040656.s003.pdf]

Figure S3: Ranked subgraphs of the beta-2-microglobulin analysis

The following figures shows the the progression of the computed score S of all distinct subgraphs of the initial beta-2-microglobulin *degradation graph*.

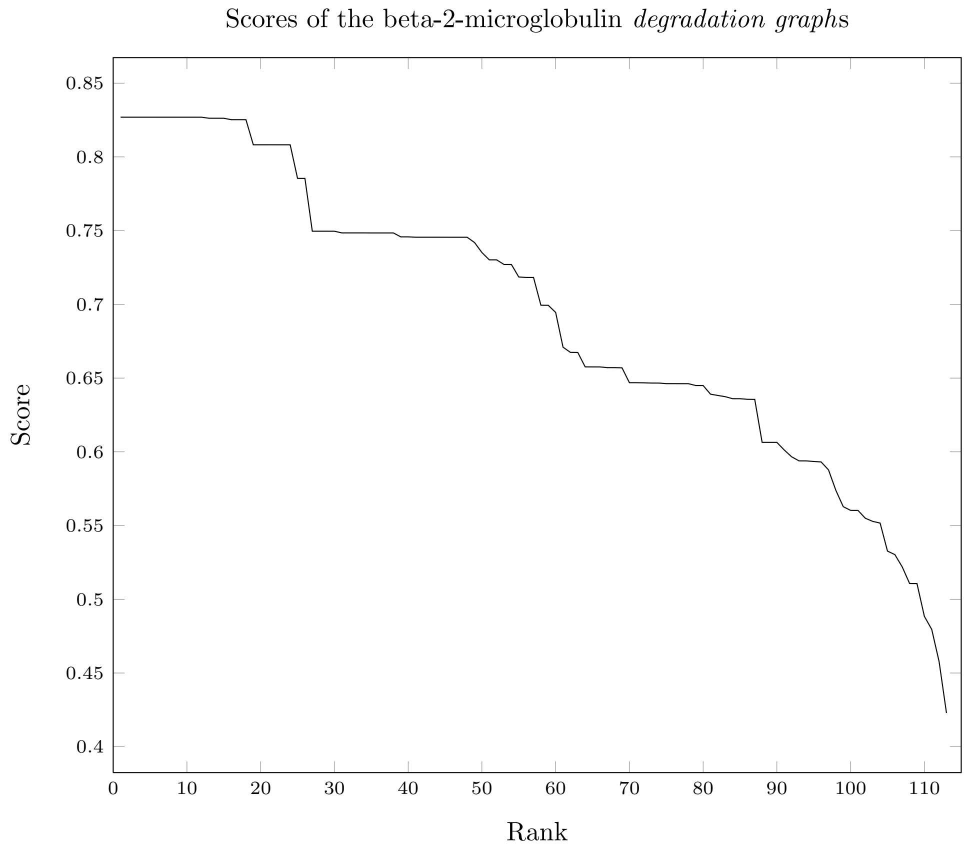

The figure clearly shows multiple areas where subgraphs were ranked with a nearly identical score (e.g., between rank 1 and 19). Reviewing the corresponding graphs, one can see, that they generally have a nearly identical structure in terms of nodes and edges. They only differ in a small number of reactions, that have mostly a reaction rate estimated to 1×10^{-6}. The bigger drops in the score can be explained by the addition or removal of nodes to the *degradation graph*.
