# Supplementary material for: Inferring Proteolytic Processes from Mass Spectrometry Time Series Data Using Degradation Graphs
Source: PLoS One. 2012 Jul 17;7(7):e40656. doi: 10.1371/journal.pone.0040656 (PMC3398944; doi:10.1371/journal.pone.0040656)
Supplement: Figure S4 — Time Courses of the confirmed beta-2-microglobulin fragments and the predicted dynamics. (PDF) [file pone.0040656.s004.pdf]

Figure S4: Time Courses of the confirmed beta-2-microglobulin fragments and the predicted dynamics.

The following figures show the time courses of all nine fragments that were confirmed manually and by our method with the observed and predicted intensity values.

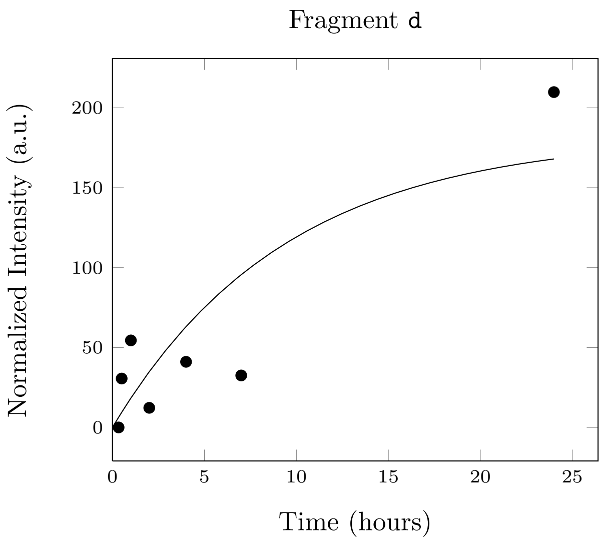
Fragment d
Normalized Intensity (a.u.)
0
50
100
150
200
Time (hours)
0
5
10
15
20
25

(a)

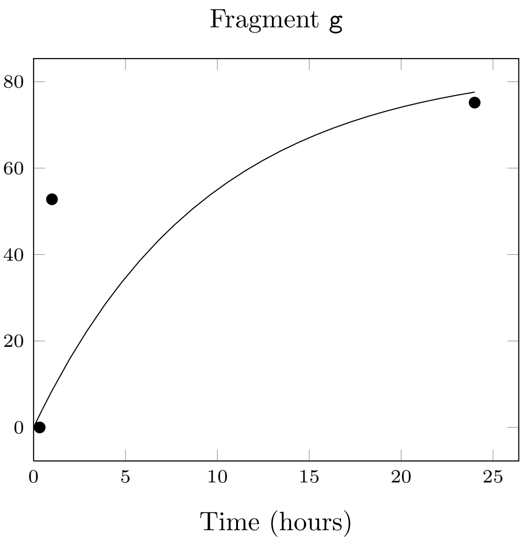
Fragment g
0
20
40
60
80
Time (hours)
0
5
10
15
20
25

(b)

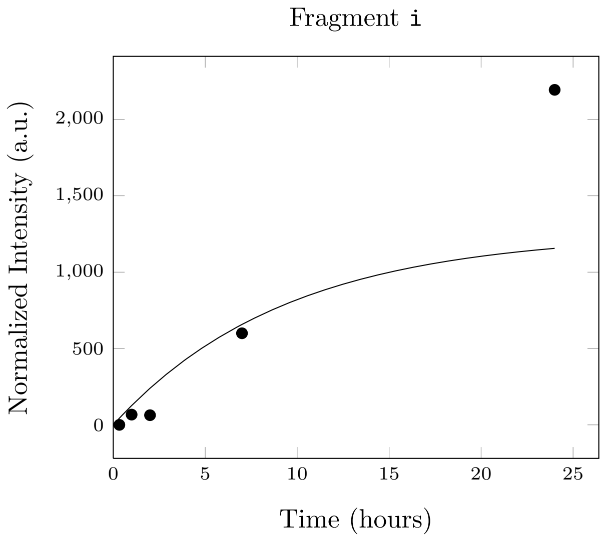
Fragment i
Normalized Intensity (a.u.)
0
500
1,000
1,500
2,000
Time (hours)
0
5
10
15
20
25

(c)

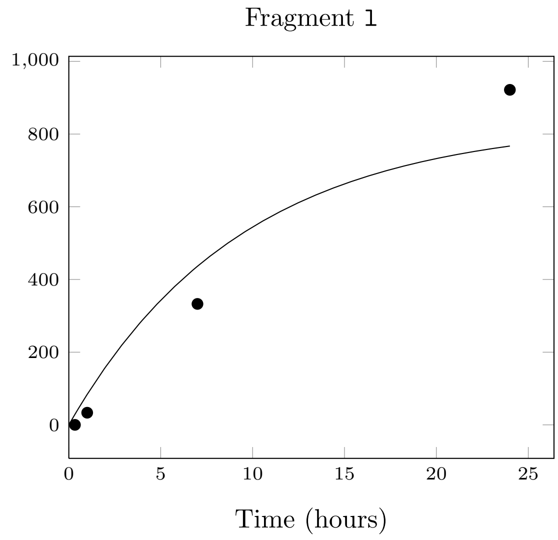
Fragment l
0
200
400
600
800
1,000
Time (hours)
0
5
10
15
20
25

(d)

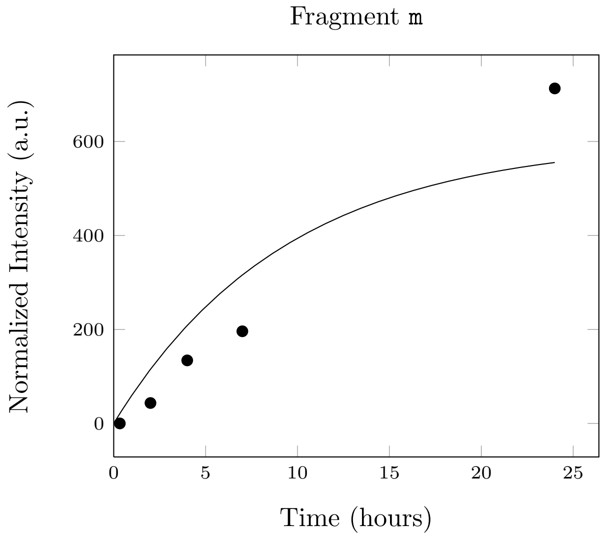
Fragment m
Normalized Intensity (a.u.)
0
200
400
600
Time (hours)
0
5
10
15
20
25

(e)

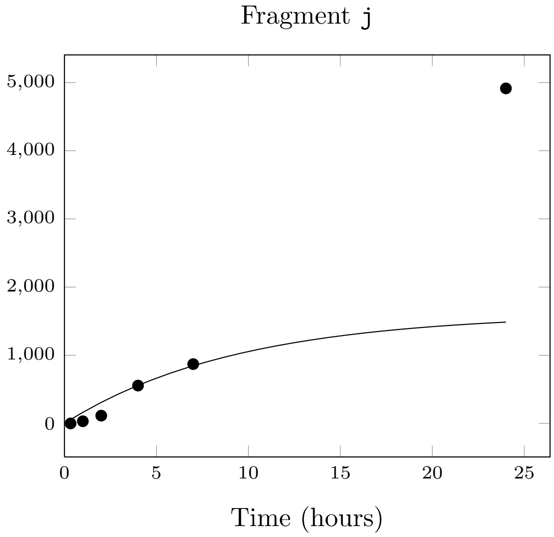
Fragment j
0
1,000
2,000
3,000
4,000
5,000
Time (hours)
0
5
10
15
20
25

(f)

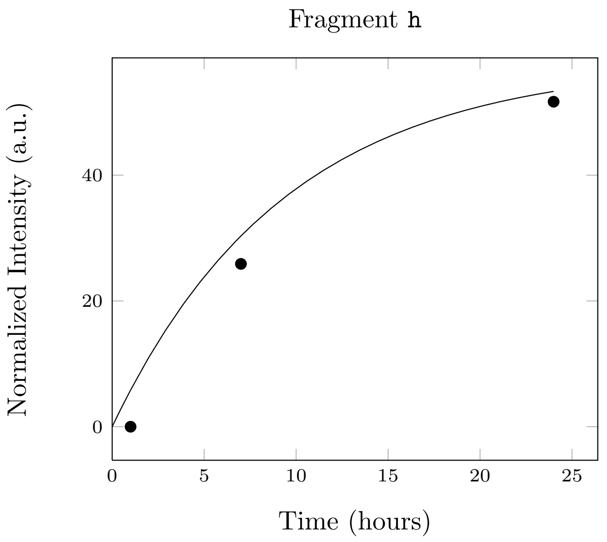
Fragment h
Normalized Intensity (a.u.)
0
20
40
0
5
10
15
20
25
Time (hours)

(a)

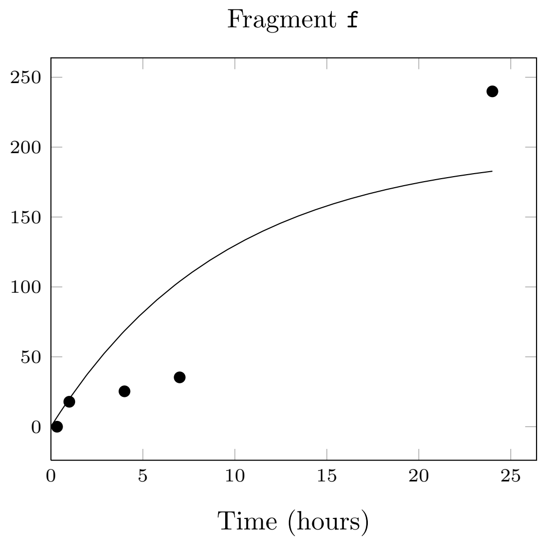
Fragment f
250
200
150
100
50
0
0
5
10
15
20
25
Time (hours)

(b)

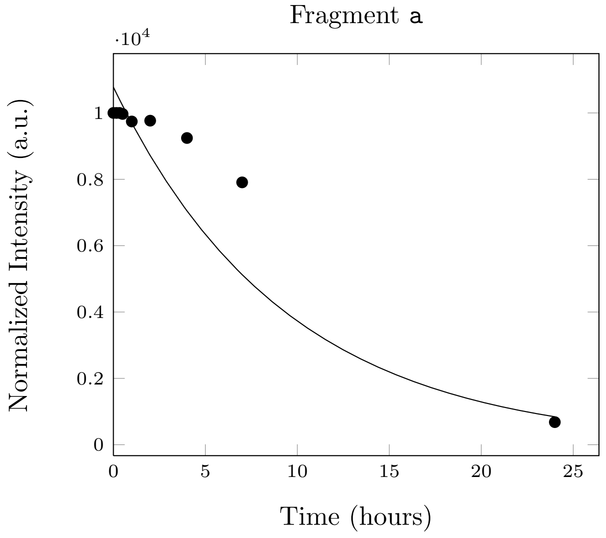
Fragment a
·10^4
Normalized Intensity (a.u.)
1
0.8
0.6
0.4
0.2
0
0
5
10
15
20
25
Time (hours)

(c)
