# Supplementary material for: Inferring Proteolytic Processes from Mass Spectrometry Time Series Data Using Degradation Graphs
Source: PLoS One. 2012 Jul 17;7(7):e40656. doi: 10.1371/journal.pone.0040656 (PMC3398944; doi:10.1371/journal.pone.0040656)
Supplement: Text S1 — A simple peptide-mass-fingerprinting (PMF) strategy to extract intensity values from mass spectra. (PDF) [file pone.0040656.s005.pdf]

Text S1: A simple peptide-mass-fingerprinting (PMF) strategy to extract intensity values from mass spectra.

In general we assume that all mass spectra were centroided prior to the analysis and that the area under the curve of the original peak was assigned to the centroided peak. We then distinguish between low and high resolution mass spectra.

For low resolution mass spectra, i.e., the isotopic peaks are not individually recognizable, but are merged into a single peak, we search for a peak with the average mass (divided by the charge) in the mass spectra. If more then one peak is found the one with the smallest distance to the theoretical average mass is chosen. The reported intensity is the intensity of the peak (i.e., the area under the curve of the original peak).

For high resolution mass spectra, i.e., the individual isotopic peaks are recognizable, we start by searching for a peak with the monoisotopic mass of the peptide. Again if more then one peak is found, we choose the one with the smallest distance. We then try to extend the isotopic pattern by searching for peaks with a distance of $+1/c$, where c is the assumed charge of the peptide. For each new peak we find we require an intensity ratio below a certain threshold ($t = 0.9$), to avoid collecting equally spaced noise peaks instead of real peptide signals.
