# Supplementary material for: Inferring Proteolytic Processes from Mass Spectrometry Time Series Data Using Degradation Graphs
Source: PLoS One. 2012 Jul 17;7(7):e40656. doi: 10.1371/journal.pone.0040656 (PMC3398944; doi:10.1371/journal.pone.0040656)
Supplement: Text S2 — Description of the normalization method used for the beta-2-microglobulin data set. (PDF) [file pone.0040656.s006.pdf]

Text S2: Description of the normalization method.

The normalization was done based on the assumption, that the sum of all intensities belonging to the proteolytic process should stay constant. Based on this we fixed the total intensity to a value of $N =$ 10.000 and distributed it over the different peaks based on their relative intensities.

$$I'_m(t) = N \frac{I_m(t)}{\sum_{j \in M} I_j(t)}$$

where $I'_m(t)$ is the normalized intensity of mass m a time point t, $I_m(t)$ the observed intensity of mass m at time point t, and M the set of all peptide masses belonging to the proteolytic process.
